# Depression among Patients with Schizophrenia in Ethiopian Mental Health Hospital: Association with Sociodemographic and Clinical Variables: A Cross-Sectional Study

**DOI:** 10.1155/2021/6697339

**Published:** 2021-02-09

**Authors:** Mandaras Tariku, Tilahun Ali, Tadesse Misgana, Mohammedamin Hajure, Henock Asfaw

**Affiliations:** ^1^Department of Psychiatry, College of Health and Medical Sciences, Haramaya University, Harar, Ethiopia; ^2^Department of Psychiatry, College of Health and Medical Sciences, Mettu University, Mettu, Ethiopia

## Abstract

**Background:**

Depression is a significant contributor to the global burden of disease and affects all individuals throughout their lifetime. Patients with schizophrenia are frequently attacked by depression during their total illness duration. Presence of comorbid depression in schizophrenia makes the patients more deteriorating and disabling course and poor outcome. *Aim of the Study*. To determine the prevalence of depression and highlight the associated sociodemographic and clinical factors in patients with schizophrenia in a specialized hospital in Addis Ababa, Ethiopia. *Setting*. This study was conducted at Amanuel Mental Specialized Hospital, Addis Ababa, Ethiopia.

**Methods:**

An institutional based cross-sectional study was conducted from May to June 2018. Depression was measured by Calgary Depression Scale for Schizophrenia on 455 samples of patient with schizophrenia and systematic sampling was used to select the study participants. Oslo Social Support Scale and Alcohol, Smoking, and Substance Involvement Screening Test were used to assess social support and substance use factors, respectively. A bivariable and multivariable logistic regression analysis model was performed to control the confounding factors. Odds ratio (OR) with the corresponding 95% confidence interval (95% CI) was determined to evaluate the strength of association.

**Results:**

A total of 445 patients responded to the questionnaire, which yields a response rate of 97.8%. The Magnitude of depression among schizophrenia patients was 24.9%. A multivariable logistic regression analysis model showed that being female [AOR 2.00, 95% CI: 1.25-3.18], divorced/widowed [AOR 2.39, 95% CI: 1.04-5.49], current substance use [AOR 1.95, 95% CI: 1.17-3.25], and poor social support [AOR 2.75, 95% CI: 1.35-5.61] were significantly associated with depression in schizophrenia.

**Conclusion:**

The magnitude of depression among schizophrenia was 24.9%. Being female, divorced/widowed, current substance use, and poor social support were associated with depression among patients with schizophrenia. Regular screening and prompt management of depressive symptoms among patients with schizophrenia is of particular importance to reduce the burden of the condition.

## 1. Introduction

Schizophrenia and depression are the severe mental disorders that are impairing the individuals' life and contributing high impacts on global burden of disease [1]. Patients with schizophrenia have high risk for developing depression compared to the general population [1–3].

Depression is frequently co-occurring among patients with mental disorder, particularly among patients with schizophrenia [4]. The risk of developing the depressive symptoms is high and ranges from 7% to 75%. In contrast to historical perspective, current evidence suggests an association of depression with poor outcomes among patients with schizophrenia [5–8].

The association of depression and psychotic symptoms has been controversial for years in the nosology of psychiatry. There is great overlap of symptoms between two conditions, particularly for the negative symptoms of psychosis, which still considered a hallmark in the diagnostic entities for some psychotic condition, like schizoaffective disorder [9, 10]. There is a notion that supports inclusion of depressive symptoms as one core features of schizophrenia syndrome from the natural history of the condition [11, 12]. Generally, it is a controversy to consider depression as a symptom of schizophrenia, comorbid symptoms, or independent condition differently unrelated to both condition [13–15].

Depressive symptoms are a part of prodrome phase and acute and postdrome phase (first six months after acute episodes) of schizophrenia [5, 16]. Globally, more than 264 million people of all ages suffer from depression and the patients with schizophrenia are more likely than the general population to experience depressive symptoms [17, 18].

The presence of depressive symptoms in schizophrenia results in the increased risk of suicide, poor quality of life, worsening the courses and outcome, exacerbate deficits in psychosocial functioning, personal suffering, and a higher rate of relapse or rehospitalization [2, 5, 13, 19, 20].

A report of previous studies shows association of depressive symptoms with the current substance used, poor social support and quality of life, family history of depressive disorder, and duration of illness in patient with schizophrenia [13–15, 21]. During the follow-up period, the medication used to treat schizophrenia, family discord, and socioeconomic problems may enhance the likelihood of developing depression among patients with schizophrenia [6, 22, 23].

Both depression and schizophrenia may have shared common risk factors that underlie the presentation of depressive symptoms in schizophrenia [11, 21]. Recognizing the contributing factors for depressive symptoms among patients with schizophrenia is significant, and early detection and treatment of comorbid depression are paramount to increase the functionality in schizophrenia [6, 14, 24].

So far, few studies have been undertaken on the magnitude of depression among patients with schizophrenia in the study area. In the current study, a reasonable amount of sample size, different in tool, that measures the depression in schizophrenia and psychosocial variables which are not addressed in the *previous studies* was considered. This has far reached the significance for different stakeholders and professionals to base the outcome of the study designing and effective psychosocial intervention strategies.

## 2. Materials and Methods

### 2.1. Study Design and Population

This study was an institutionalbased cross-sectional study carried out among patients with schizophrenia who are following their medications in Amanuel Mental Specialized Hospital. Adult patients with schizophrenia were included in the study, and patients who are acutely ill and with intellectual disability were excluded from the study.

### 2.2. Sampling Technique

Averagely, there were more than 3420 patients visited outpatient departments monthly. Ever since, there were difference in numbers of patients with schizophrenia who are following their mental health care service in the outpatient department; the samples were distributed to all eight OPD through proportional allocation. Finally, a systematic sampling technique was used to select the representative samples from the outpatient departments based on the interval of 8.

### 2.3. Study Instrument

Five different categories of measures were used in this study. Firstly, instruments are related to sociodemographic characteristics, such as age, sex, marital status, occupation, and educational status. Secondly, clinical characteristics include age at the first onset of illness, duration of treatments, previous hospitalization, and number of psychotic episode. Thirdly, social support was measured by Oslo Social Support Scale. The score between 3 and 8 is poor social support, 9 and 11 is moderate social support, and 12 and 14 is strong social support [25]. The internal consistency of Oslo Social Support Scale was 0.50 among Nigerian clinical students [26]. The tool has been used in population level in Ethiopia [26]. Fourthly, Alcohol, Smoking, and Substance Involvement Screening Test (ASSIST) was used to measure the current and lifetime use of substances. Lastly, depression was measured by using Calgary depressive scale for schizophrenia. It has 9 items of self-reported measures. The score is rated 0 (absent), 1 (mild), 2 (moderate), and 3 (severe). Based on this scale, score more than six [6] had used to determine the prevalence of depression among patients with schizophrenia [27, 28]. The Calgary depression scale for schizophrenia has high interrater reliability and a valid measure of depression among patients with schizophrenia [29]. The reliability test was done on the scale to assess depression among schizophrenia, and the Cronbach's alpha was 0.88.

### 2.4. Data Management and Analysis

Data were checked for completeness and consistency. Then it was coded and entered by using Epidata version 3.1 and analyzed by using Statistical Package of Social Science (SPSS) version 20. Binary and multivariable logistic regression was used to assess the factors associated with depression among patients with schizophrenia. AOR with 95% CI and *p* value less than 0.05 was considered statistically significant.

### 2.5. Ethical Consideration

Approval for this study was obtained from the joint ethical review committee of the University of Gondar and AMSH, with reference number AMSH/146/4/225. International ethical norms and standards were strictly adhered to at all times. Informed written consent was obtained from participants. Verbal and written consents were obtained from all the participants. Participation was voluntary.

## 3. Results

### 3.1. Socio-demographic Characteristics of the Study Participants

A total of 445 patients responded to the questionnaire. The mean age of the respondents was 35.92 ± 7.9 years. The majority of the participants were male 275 (61.8%), 211 (47.4%) were orthodox in religion, 49.9% were single, 45.8% were able to read and write, and 36.9% were jobless (Table 1).

### 3.2. Clinical Characteristic of the Study Participants

From a total of 445 participants, 264 (59.3%) had more than two psychotic episodes in patients with a diagnosis of schizophrenia and 238 (53.5%) had a previous history of hospitalization. The mean age of the first onset of schizophrenia was 25.7 ± 5.2 years, and the mean duration of treatment was 6.64 ± 3.51 years (Table 2).

### 3.3. Social Support Characteristics of the Study Participants

Regarding to social support, the majority of respondents had poor social support 264 (59.3%), 74 (16.6%) had moderate social support, and 107 (24.0%) had strong social support.

### 3.4. Lifetime and Current Substance Use among the Respondents

Regarding to substance use, half of the respondents used any substance in their lifetime and 196 (44%) of the respondents used any substance of abuse in the past three months before data collection.

### 3.5. The Magnitude of Depressive Disorder among the Patients with Schizophrenia

**Figure 1:**
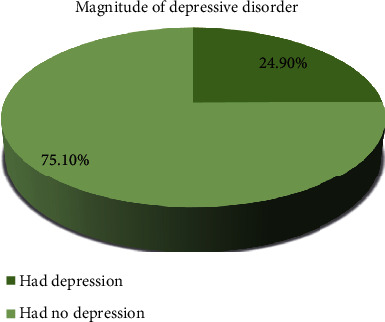
Magnitude of depressive disorder among the patients with schizophrenia in AMSH 2018.

The magnitude of depressive disorder among the patients with schizophrenia was 24.9% with 95% CI 21.3-28.8 (Figure 1).

### 3.6. Association of the Independent Variables with Depression

Table 3 summarizes the results of bivariate and multivariable analysis for associations with depression. In bivariate analysis, sex, marital status, poor social support, using a typical antipsychotic medication, previous hospitalization, two or more episodes of psychotic symptoms, and current substance use were the significant association with depression among patients with schizophrenia. Finally, the results of multivariable logistic regression analysis showed that female sex [AOR = 2, 95% CI: 1.25-3.18], current use any substances [AOR = 1.95, 95% CI: 1.17-3.25], poor social support [AOR = 2.75, 95% CI: 1.35-5.61], and being divorced and widowed [AOR = 2.39, 95% CI: 1.04-5.49] were found to be the significant predictors of depression among the patients with schizophrenia (Table 3).

## 4. Discussion

The patients with schizophrenia have a higher risk of developing depression. The presence of depression among patients with schizophrenia is associated with poor prognosis and outcomes compared to patients with schizophrenia without depression.

According to this study, the magnitude of depression among patients with schizophrenia was 24.9% (95% CI: 21.3-28.8). This finding is consistent with a cross-sectional study conducted in Turkey (26.5%) [14]. On the other hand, the finding of the current study was higher than that of the study done in the USA (20%) [30]. The possible reason for the discrepancy might be the difference in a measurement tool in which Hamilton Depression Rating Scale was used to measure depression in the USA.

The result is low compared with that of a cross-sectional study conducted in France (30%) [31], Germany (36%) [10], and China (54.6%) [32]. The discrepancy might be due to psychosocial support level, measurement difference, and differences in sensitivity and specificity of the measure of depression among patients with schizophrenia in a study conducted in China.

This finding is further supported and acknowledges that the prevalence of depressive disorder is twofold higher among women than among men. Accordingly, the odds of having depressive symptoms among female participants were about 2 times higher compared with those among male participants [AOR = 2, 95% CI: 1.25-3.18]. The finding is consistent with a large representative population-based study (*n* = 89037) [21]. This might be due to hormonal differences, the effects of childbirth, differing psychosocial stressors for women and for men, and behavioral models of learned helplessness.

On the other hand, there were inconsistent result with a cross-sectional study conducted in China [15] which showed that being male was associated with depression in patient with schizophrenia. Another study did not show a significant gender difference in depressive symptoms among schizophrenia population. This variability is due to the distribution of study participants and the nature of the course of illness.

The odds of having depressive disorder among participants who used any substance in the past three months were about 1.95 times higher compared with those among participants who did not use any substance in the past three months [AOR = 1.95, 95% CI: 1.17-3.25]. The finding is consistent with a cross-sectional study conducted in Ethiopia [23]. The reason could be justified by the direct effect of the substance on the brain and the psychosocial problems related to using the substance.

The study participants with poor social support were 2.75 times more likely to have depression compared with participants with strong social support [AOR = 2.75, 95% CI: 1.35-5.61]. This could be justified by poor social support which is one of the determinant factors among patients with mental disorders to develop co-occurring of depressive disorder [33]. The studies indicate that social support is essential for maintaining physical and psychological health [34]. The finding further supports the recognition of psychosocial determinant for the causation of depressive disorder [33, 35, 36].

Finally, patients who had divorced/widowed were 2.39 times more likely to be depressed compared to those who had married [AOR = 2.39, 95% CI: 1.04-.5.49]. The finding is agreement with a previously conducted study in Ethiopia [23]. This might be due to the effect of marital disruption associated with mental health problems and the psychosocial impact of loneliness [24]. The reports previously conducted on psychological aspects of divorced/windowed revealed that divorce and death of the spouse appear to affect both the sexes in different ways and negatively affect the mental health status of the persons [37].

## 5. Limitation of the Study

Due to its cross-sectional nature, the study could not explore the cause and effect relationship between outcome and independent variables. The presence of chronic medical conditions, duration of illness, and positive and negative symptoms of schizophrenia were not assessed that could affect the magnitude of depression and associated factors among patients with schizophrenia.

## 6. Conclusion and Recommendation

The aim of this study was to assess the magnitude and associated factors of depression among patients with schizophrenia. Accordingly, the magnitude of depression was 24.9%. Being female , divorced/widowed, current substance use, and poor social support were associated with depression among patients with schizophrenia. Regular screening and prompt management of depressive symptoms among patients with schizophrenia are of particular importance to reduce the burden of the condition.

## Figures and Tables

**Table 1:** Description of sociodemographic characteristics of the patients with schizophrenia in AMSH 2018.

Variable	Category	Frequency	Percentage
Age mean (SD)		35.92 (7.90)	
Sex	Male	275	61.8
	Female	170	38.2
Marital status	Single	222	49.9
	Married	144	32.4
	Separated	45	10.1
	Divorced/widowed	34	7.6
Ethnicity	Amhara	170	38.2
	Oromo	131	29.4
	Tigre	40	9.0
	Gurage	91	20.4
	Others^∗^	13	2.9
Religion	Orthodox	211	47.4
	Muslim	179	40.2
	Protestant	42	9.4
	Others^∗∗^	13	2.9
Occupation	Government employed	88	19.8
	Merchant	47	10.6
	Farmer	47	10.6
	Student	49	11
	Day laborer	29	6.5
	House wife	21	4.7
	Jobless	164	36.9
Educational status	Unable to read and write	142	31.9
	Able to read write	204	45.8
	Primary	43	9.7
	High school	24	5.4
	College and above	32	7.2

Others^∗^: Silte and Wolayta; Others^∗∗^; catholic and Waaqeffanna.

**Table 2:** Description of the clinical characteristic of the patients with schizophrenia in AMSH 2018.

Variables	Category	Frequency	Percentage
Age at first onset of illness mean (SD)	25.7 (5.2)		
Duration of treatment median (IQR)	6 (6)		
Previous hospitalization	Yes	238	53.3
	No	207	46.5
Number of psychotic episodes	First episode	181	40.7
	≥2 episodes	264	59.3

**Table 3:** Factors associated with depression among the patients with schizophrenia in multivariable logistic regression.

Explanatory variables	Category	Depressive symptoms		COR (95% CI)	AOR (95% CI)
		Yes (*N*)	No		
Sex	Male	54	221	1	1
	Female			2.06 (1.34-3.19)	2.00 (1.25-3.18)^∗∗^
Marital status	Single	50	172	0.98 (0.59-1.61)	0.79 (0.46-1.36)
	Separated	10	35	0.96 (0.43-2.15)	0.60 (0.25-1.45)
	Divorced/widowed	18	16	3.78 (1.74-8.23)	2.39 (1.04-5.49)^∗^
	Married	33	111	1	1
Type of antipsychotic	Typical	63	136	1.90 (0.87-4.19)	1.53 (0.65-3.58)
	Atypical	39	161	1.00 (0.44-2.23)	1.00 (0.42-2.36)
	Both	9	37	1	1
Current substance use	Yes	69	127	2.68 (1.72-4.17)	1.95 (1.17-3.25)^∗^
	No	42	207	1	1
Previous hospitalization	Yes	74	166	2.02 (1.29-3.17)	1.23 (0.71-2.14)
	No	37	168	1	1
Number of psychotic episodes	Single episode	32	154	1	1
	≥2 episodes	79	180	2.11 (1.33-3.36)	1.58 (0.93-2.66)
Social support	Poor	82	182	3.57 (1.85-6.86)	2.75 (1.35-5.61)^∗∗^
	Moderate	17	57	2.36 (1.05-5.30)	1.98 (0.82-4.77)
	Strong	12	95	1	1

^∗^ *p* < 0.05 and ^∗∗^*p* < 0.01; chi square = 6.46; DF = 8; Hosmer-Lemeshow test = 0.60.

## Data Availability

The datasets used and/or analyzed during the current study available from the corresponding author on reasonable request.

## Abbreviations

AMSH: Amanuel Mental Specialized Hospital
ASSIST: Alcohol, Smoking, and Substance Involvement Screening Test
AOR: Adjusted Odd Ratio
SD: Standard Deviation
SPSS: Statistical Package of Social Science
CI: Confidence Interval
WHO: World Health Organization.
